# Adipose-Derived Stem Cells Secretome and Its Potential Application in “Stem Cell-Free Therapy”

**DOI:** 10.3390/biom11060878

**Published:** 2021-06-13

**Authors:** Anna Trzyna, Agnieszka Banaś-Ząbczyk

**Affiliations:** Department of Biology, Institute of Medical Sciences, Medical College of Rzeszow University, 35-959 Rzeszow, Poland; a.a.trzyna@gmail.com

**Keywords:** adipose-derived stem cells, extracellular vesicles, secretome, stem cell therapy

## Abstract

Adipose-derived stem cells (ASCs) secrete many cytokines, proteins, growth factors, and extracellular vesicles with beneficial outcomes that can be used in regenerative medicine. It has great potential, and the development of new treatment strategies using the ASCs secretome is of global interest. Besides cytokines, proteins, and growth factors, the therapeutic effect of secretome is hidden in non-coding RNAs such as miR-21, miR-24, and miR-26 carried via exosomes secreted by adequate cells. The whole secretome, including ASC-derived exosomes (ASC-exos) has been proven in many studies to have immunomodulatory, proangiogenic, neurotrophic, and epithelization activity and can potentially be used for neurodegenerative, cardiovascular, respiratory, inflammatory, and autoimmune diseases as well as wound healing treatment. Due to limitations in the use of stem cells in cell-based therapy, its secretome with emphasis on exosomes seems to be a reasonable and safer alternative with increased effectiveness and fewer side effects. Moreover, the great advantage of cell-free therapy is the possibility of biobanking the ASCs secretome. In this review, we focus on the current state of knowledge on the use of the ASCs secretome in stem cell-free therapy.

## 1. Introduction

Mesenchymal stem cells (MSCs) have a great potential for use in medicine and stem cell therapy. The therapeutic potential of MSCs is mainly based on their ability to self-renew and differentiate towards tissue-specific cells, thus tissue regeneration [1,2]. To standardize the concept and the possibility of comparing the results of research in possible medical application, in 2006, the International Society for Cellular Therapy (ISCT) published three minimum criteria for defining MSC cells. According to ISCT, these cells must be plastic-adherent, capable of differentiating into osteoblasts, adipocytes and chondroblasts, and phenotypically be ≥95% CD105, CD90, and CD73 positive and negative for CD45, CD34, CD14 or CD11b, CD79alpha or CD19 and HLA-DR [3]. MSCs can be hypothetically obtained from almost all human tissues, e.g., the marrow spaces of long bones, cord blood, dental pulp, placenta, and synovial fluids, first discovered and described by Friedenstein’s group (1976), Muluen’s group (1978), Shi’s group (2003), and Atala’s group (2006), respectively [4,5,6,7]. However, due to many limitations in isolation and abundance, the main sources today are bone marrow (BM) and adipose tissue (AT) [2,8]. BM-MSCs were the first discovered and described at the end of the twentieth century, and naturally, BM became the first and most important source of MSCs [8]. However, subsequent researchers provided evidence that multipotent stem cells can be successfully isolated from other tissues and that higher densities of MSCs can be found in AT than BM. At the same time, the invasiveness of donor sampling is reduced [9].

Adipose-derived stem cells are mesenchymal stem cells harvested from AT capable of self-renewal and multipotent differentiation towards adipocytes, osteoblasts, chondrocytes, myocytes, neurocytes, and other types of cells [10,11,12,13,14]. ASCs isolated from AT meet the criteria established by the ISCT and are considered a promising tool for use in regenerative medicine, including the treatment of degenerative, inflammatory, and autoimmune diseases [13]. ASCs have already been shown to have the potential for use in multiple sclerosis [14], rheumatoid arthritis, osteoarthritis [13,15,16,17,18], fistulae [18,19], diabetes mellitus [20,21], dyslipidemia, and cardiovascular diseases [22,23] treatment as well as skin aging and wound healing [24,25,26]. The great advantage of MSCs isolated from AT is that they can be easily obtained by liposuction from autologous subcutaneous adipose tissue with high cellular activity without any ethical concerns comparing to embryonic stem cells, the obtainment of which is associated with the destruction of the embryo. This makes ASCs a suitable choice for use in cell-based therapies, apart from those that may be questionable and controversial [10].

ASCs may also be considered a therapeutic tool due to their autocrine and paracrine factors and by increasing the recruitment of endogenous precursors. They secrete many significant proteins, including growth factors (GF) and cytokines [27,28,29], as well as extracellular vesicles (EV) and RNAs [30,31,32] to support cell regeneration, proliferation, differentiation, and migration [33]. Attempts have been made to optimize the conditions of stem cell-based therapy with naive ASCs by manipulating the route of administration and dose of cells and the timing [34,35,36] of homing to the site of inflammation, however, without fully satisfactory results [37,38]. The Sacerdote group has been studying the use of ASCs for years [34]. In 2013, they showed that 1 × 10^6^ human ASCs administered intravenously can reduce pain in a mouse model with neuropathic pain. However, they suggested validation of ASCs dose and the time of treatment to improve this effect [35]. In a clinical study by Alvaro-Gracia et al., after three months of treatment of the rheumatoid arthritis patients (three groups: 1, 2, and 4 million cells/kg by three intravenous administrations on days 1, 8, and 15), clinical benefits were not sustained but many side effects were observed [36]. Therefore, a new strategy for ASCs based therapy is needed, and ASCs secretome can be a promising tool for medical purposes as a safe therapeutic agent for cell-free-based therapy with easy storage for long-term use [39,40].

This review provides information on the current state of knowledge about secretome of adipose-derived stem cells, with particular focus on their exosomes. The study used all available sources such as PubMed, Google Scholar, and Clinical Trials (European and World) databases. The authors collected data and summarized information from in vitro, in vivo, and clinical trials using adipose-derived stem cells secretome in the context of use in stem cell-free therapy. The literature study was based on keywords such as adipose-derived stem cells, secretome, exosomes, growth factors, cytokines, cell-free, therapy that were used alone or in combination with diabetes mellitus, cardiovascular, wound healing, neurodegenerative, skeletal, respiratory, metabolic, regeneration, disease, and skin aging.

## 2. Adipose-Derived Stem Cells Characteristics

### 2.1. Nomenclature

The International Fat Applied Technology Society has proposed and adapted the term adipose-derived stem cells (ASCs) to standardize it to avoid confusion in nomenclature. In the older literature, adipose-derived stem cells are referred to by various terms and abbreviations: adipose-derived adult stem (ADAS) cells; adipose mesenchymal stem cells (AdMSCs); adipose-derived adult stromal cells; adipose-derived stromal cells (ADSCs); adipose stromal cells (ASCs); adipose-derived stromal/stem cells (ASCs); vascular stromal/stem cells; preadipocytes; processed lipoaspirate (PLA) cells; pericytes and lipoblasts and that inconsistent nomenclature in the literature has led to confusion [41,42,43,44]. In line with International Fat Applied Technology Society recommendations, we use the term adipose-derived stem cells in this study.

### 2.2. Sources

Adipose-derived stem cells can be harvested from adipose tissue also containing adipocytes, preadipocytes, pericytes, and immune cells such as eosinophils, macrophages, and innate lymphoid cells (ILCs), T cells, and B cells, fibroblasts, and endothelial cells [45,46]. Before International Fat Applied Technology in 2004 defined the nomenclature and adapted the term ASCs with a specific phenotype, referring only to mesenchymal stem cells isolated from the vascular stromal fraction, the terms preadipocytes and pericytes had previously been used confusingly [41,42,43]. Adipose tissue can be divided into white (WAT) and brown (BAT) adipose tissue as a site for energy storage and expenditure, respectively. Adipose-derived stem cells from WAT are characterized by a different expression of cell surface markers than BAT, e.g., ASCs from BAT express noticeably more TMEM-26, SSEA-4, CD106, CD105, HLA-A,B,C, and CD-137 but less CD86 and LIN, than WAT [47]. Subcutaneous AT is most commonly used for ASCs isolation due to its easy and non-invasive obtaining procedure from the abdomen, thigh, and arm. [48,49]. Moreover, those obtained from thighs show the best viability [50]. However, it can also be obtained from the craniofacial, pericardial, perirenal, omentum, intestine, bone marrow, buttocks, pulmonary (WAT regions) cervical, paravertebral, supraclavicular, axillary, suprarenal (BAT regions) regions [43,49,51]. The type of harvested area and the type of adipose tissue affects proliferation, endocrine function, gene expression, surface antigens, and the differentiation potentials of adipose-derived stem cells [50,52]. Nepali et al. have already shown that abdominal ASCs exhibit better chondrogenic but poorer osteogenic potential than orbital ASCs [53].

Moreover, the origin of the adipose-derived stem cells is critical, and the age, weight, and disease state of the donor may affect the condition and properties of isolated ASCs. It has been shown that the older donor of ASCs the lower proliferation and differentiation potential and the lower growth factors secretion ability [9,36,54]. Furthermore, ASCs obtained from obese donors are characterized by decreased expression of phenotypic mesenchymal stem cells markers, an excessive immune response, and thus a lower self-renewal, differentiation potential [55,56], and a higher capacity for migration, invasion, and phagocytosis [57]. Studies have also shown that type 1 and type 2 diabetes mellitus, hypercholesterolemia, hypertension, and smoking have negative effects on the pluripotency and self-renewal of isolated ASCs [56,58]. On the other hand, it has been shown that gender and chemotherapy have not shown much effect on ASCs [59]. However, it is necessary to use exosomes isolated from healthy individuals to avoid a reduction in the therapeutic potential and potential side effects caused by bioactive exosomes, the content of which depends on the state of secretory cells, therefore their pathological effects should be assessed [60].

### 2.3. Phenotype

Adipose-derived stem cells are a heterogenic population and no unique surface markers have yet been described. However, they express markers characteristic of mesenchymal stem cells established by ISCT and the International Federation for Adipose Therapeutics and Science (IFATS) and are described by CD73(+), CD90(+), CD105(+), and CD36(+) but also CD31(−), CD45(−), CD11b(−), CD106(−). The expression of CD36 and lack of expression of CD106 distinguish ASCs from BM-MSCs [61]. Moreover, ASCs express β-1 the integrin (CD29) that participates in angiogenesis and CD44 hyaluronate and osteopontin receptor, which is crucial in extracellular matrix development and pathological processes such as neoplasia [62,63]. Although, they also show the characteristics of neural phenotypic profile through the expression of NEUROD1, SOX3, and PAX6 and markers determining core circuitry self-renewal: SOX2, NANOG, OCT4 [64]. There are inconsistent data, confounding if ASCs isolated from stromal vascular fraction may express CD34 [65,66] and CD106 [67] or not [68]. It is recognized that cultured MSCs do not express CD34 contrary to freshly isolated cells. Some studies have shown that CD34 is present at the beginning of culture in freshly isolated ASCs but after the first and subsequent passages (2–4) disappears [69] or remains at a low level [70]. 

ASCs markers also depend on adipose tissue origin, and it has been proven that orbital-ASCs express less HLA-DR, CD31, and CD44 than abdominal-ASCs [53]. It has also been shown that the ASCs phenotype changes progressively with passages in vitro. Stromal-cell associated markers such as CD13, CD29, CD44, CD63, CD73, CD90, CD166 were present after subsequent passages but with increased levels [68,70]. Zhu et al. have also shown that ASCs, retained their phenotype after 25 passages, as well as multilineage differentiation, and proliferation capacity but decreased compared to the first passage [71]. Furthermore, Atat et al. confirmed that the expansion of the passaged ASCs did not influence their differentiation capacity [72]. Interestingly, Griffin et al. showed that the phenotype and differentiation potential are not changed by comparing ASCs from healthy and systematic sclerosis donors but only the proliferation, invasion, and migration capacity of these cells, which may suggest that the diseases does not alter the self-renewal potency of ASCs [73]. Similarly, Mieczkowska et al. showed that ASCs from oncological surgery patients exhibit comparable phenotypes to those from healthy donors [70].

## 3. Adipose-Derived Stem Cells Secretome

**Figure 1:**
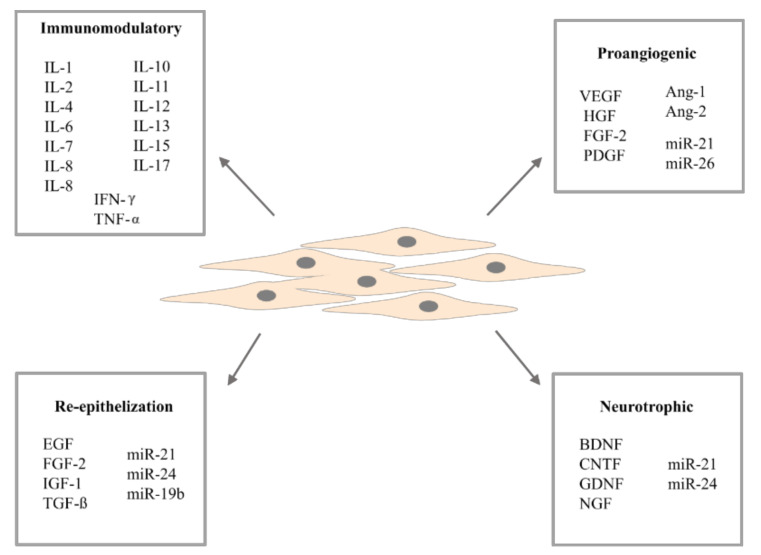
Different types of secretory activity of adipose-derived stem cells.

Adipose-derived stem cells produce many molecules responsible for cell signaling, such as cytokines [29,74], growth factors [28,75], morphogens [76], chemokines [27,77], and extracellular vesicles [78,79,80], improving various cellular mechanisms. Interestingly, compared to BM-MSCs—the second most frequently considered MSCs for stem cell therapy—ASCs secrete more growth factors, and this feature increases their regenerative capacity [2,81]. It has also been shown, in vivo, that ASCs are better in the bioactive factors secretion such as nerve growth factor (NGF), hepatocyte growth factor (HGF), monocyte chemotactic protein 1 (MCP-1), colony stimulator of granulocyte-macrophage factor (GM-CSF), granulocyte colony stimulating-factor (CSF), interleukin 1 receptor antagonist (IL-1RA), interleukin (IL)-6 and IL-8 versus bone marrow (BM)-MSCs [27]. This implies a better differentiation, migration, proliferation, and autocrine activity compared to BM-MSCs; moreover, an excellent ASCs paracrine potential has been revealed. Additionally, ASCs undergo senescence later than BM-MSCs [82,83]. Due to several limitations of cell therapy, in terms of increasing the effectiveness of ASCs and in view of safety and costs, the dose and frequency of cell application cannot be increased indefinitely. One strategy for enhancing ASCs therapy is to use their secretome with particular emphasis on extracellular vesicles. This approach promises higher efficiency than naive ASCs without having to use ASCs alone [37] (Figure 1).

### 3.1. Cytokines and Growth Factors

Adipose-derived stem cells are characterized by high trophic activity and secretion of a large amount of proteins, growth factors, and pro- and anti-inflammatory cytokines exerting benefits towards cells’ regenerative capacity. They are considered to be highly immunomodulating cells, exceeding the suppressive effect of BM-MSCs by secreting more anti-inflammatory IL-6 and transforming growth factor-β1 (TGF-β1) [84]. However, under different conditions, IL-6 can have anti- and pro-inflammatory properties similar to IL-2 [77]. It has been shown that relevant levels of IL-2 affect ASCs function by transcriptional dysregulation [85] and also that IL-6 enhances ALP activity and promotes osterix expression, and thus osteogenesis [86,87]. Moreover, it has been shown that orbital ASCs secrete higher concentrations of IL-6, IL-8, eotaxin, fractalkine, and IL-10, but lower concentrations of basic fibroblast growth factor (FGF-2) and vascular endothelial growth factor (VEGF) than abdominal ASCs [53]. In addition, high seeding density, long-term culturing, and passaging in vitro reduce the concentration of IL-6 and VEGF [72,88].

In addition to bifunctional IL-2 and IL-6, adipose-derived stem cells secrete other cytokines with a well-defined pro-inflammatory (IL-7, IL-8, IL-9, IL-11, IL-12, IL-15, IL-17, interferon-gamma IFN-γ, IL-1β, and tumor necrosis factor-alpha TNF-α) or anti-inflammatory (IL-1Ra, IL-4, IL-10, and IL-13) properties [29,44,89]. It has been shown that TNF-α and IL-1β secretion by macrophages mediates the inhibitory effect on ASCs adipogenesis, and also that combination of TNF-α or IL-1β with IFN-γ can enhance the immunomodulatory properties of ASCs mainly dependent on indoleamine 2, 3-dioxidase (IDO) or inducible nitric oxide synthase (iNOS) [74]. IFN-γ triggers ASCs to elicit immunosuppressive factors [90]. In addition, the signaling protein (PGE2) secreted by ASCs has been found to have an immunosuppressive effect [91,92]. It has been shown that IL-4 and IL-17 have an inhibitory effect on adipogenic differentiation by promoting lipolysis and suppressing proadipogenic factors gene expression, respectively [93,94].

In addition to cytokines, ASCs secrete many growth factors that influence cellular processes that promote regeneration. The proangiogenic and antiapoptotic properties of ASCs are provided by trophic factors secreted by these cells such as VEGF, FGF-2, TGF-β, HGF, and GM-CSF [95]. HGF and VEGF also induce neurogenic responses [33]. Interestingly, the secretion of HGF involved in vasculogenesis and angiogenesis [96] is significantly increased after ASCs stimulation with FGF-2 or epidermal growth factor (EGF) [29]. However, platelet-derived growth factor (PDGF) secreted by ASCs plays an essential role in angiogenesis [97] and it has been shown that cell stimulation with PDGF enhances the release of extracellular vesicles and thus proangiogenic properties [98]. Adipose-derived stem cells also secrete insulin-like growth factor (IGF) promoting, proliferation, self-renewal, and differentiation of cells [99] but its level decrease with donor age [100]. Nevertheless, IGF-1, EGF, FGF-2, and TGFα are essential wound healing factors, enhancing these processes and cell migration [101].

### 3.2. Extracellular Vesicles

**Figure 2:**
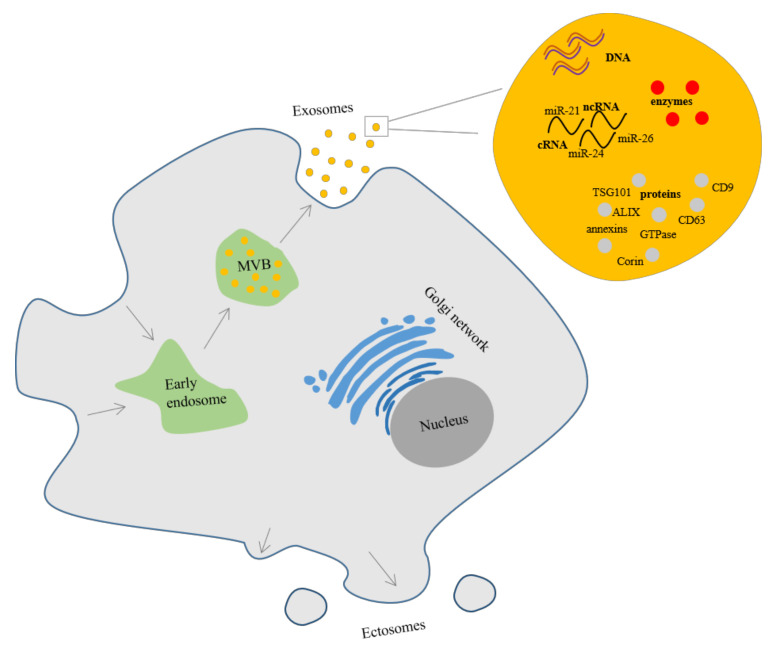
Exosomes and ectosomes secretion in adipose-derived stem cell.

Extracellular vesicles (EV) are secreted by cells into the extracellular matrix and carry biomarkers that influence numerous cellular processes. These self-contained lipid bilayer vesicles are composed of lipids, proteins, and nucleic acids [102], but cannot replicate because they do not contain a functional nucleus [103]. EV can be formed by outward budding of the cell membrane or from inward endosome. Exosomes are formed from exosome precursors called intraluminal vesicles (ILVs) in the endocytic cisternae membrane. ILVs accumulation leads to the formation of multivesicular bodies (MVBs), which over time exocytically fuse with the plasma membrane and can be released into the extracellular space. Whereas, ectosomes are quickly generated in the plasma membrane (Figure 2) [104]. The term nanovesicles can also be found in the literature. Nanovesicles are membrane-bounded structures of various origins, e.g., exosomes, so basically exosomes are nanovesicles. Nanovesicles can be artificially generated by sequential cell membrane penetration and can be used in cell-based therapy [105].

EVs have attracted a lot of attention over the last decade and there has been a growing number of scientific papers on them. There is no consensus yet on the description of specific markers for EVs subtypes. Due to this, the International Society for Extracellular Vesicle published in 2018 an updated minimum information statement on extracellular vesicles (MISEV2018) referring to meaningful changes in nomenclature. MISEV2018 recommends to describe subtypes of EVs in published researches by (a) physical characteristics such as density (low, middle, high, with each range defined) or size (“small EVs” (sEVs): <100 nm or <200 nm and “medium/large EVs” (m/lEVs): >200 nm (large and/or medium)) (b) biochemical composition (CD63+/CD81+-EVs, Annexin A5-stained EVs, etc.); or (c) descriptions of cell conditions or origins (podocyte EVs, hypoxic EVs, large oncosomes, apoptotic bodies). However, using subtype terms based on subcellular origin is allowed: endosome-origin “exosomes” and plasma membrane-derived “ectosomes” (microparticles/microvesicles), while a demonstration of origin is shown [106].

#### 3.2.1. Extracellular Vesicles Composition

Adipose-derived stem cells secrete exosomes (Figure 2) with a diameter of 30–100 nm [30] or according to other sources up to 150 nm [107,108]. Exosomes contain cytoskeleton, heat shock, and transmembrane proteins, enzymes, lipids, and DNA [102]. Moreover, they carry coding (mRNA) and non-coding RNA (e.g., rRNA, miRNA) in various subtypes but with a strong emphasis on short non-coding RNA [109,110]. Non-coding RNAs are RNA molecules that cannot be translated into a protein and the functions of many have not been understood yet, however, carried by exosomes play a role in cellular mediated communication [110]. Interestingly, Xing et al. identified 1185 proteins in ASC-derived exosomes (ASC-exos) and showed that exosomes are crucial not only for cell-to-cell communication but also for metabolic and cellular processes and the regulation of biological processes. They also showed that ASC-exos are characterized by exosomal markers such as CD9, CD63, as well as tumor susceptibility gene (TSG) 101 [111]. In turn, Ni et al. identified 1466 proteins in ASCs-exos and some of them are associated with the proliferation and regeneration pathways (e.g., PI3K-Akt or WNT) such as LAMC1, LAMA4, LAMB2, LAMB1, MEK1, MEK2, IKBKA, IKKA CHUK, RELN, VTN, WNT2, PEDF, and SERPINF1 [112]. Interestingly, González-Cubero et al. showed that exosomes derived from ASC-conditioned medium were characterized by a strong enhancement of CD3, CD45, CD56, HLA-ABC, and HLA-DRDPDQ expression [113].

#### 3.2.2. Control of the Secretion of Extracellular Vesicles

The specific molecule content makes exosomes (due to their specific biological properties) modulators with different effects on the target recipient cells. ASC-exos provide benefits in therapeutic use due to their stability in the human body [30] as opposed to whole ASCs which die after 48 h of systemic infusion [38]. Moreover, the use of exosomes is safer, because of the reduced risk of tumorigenicity. They can also be readily produced in large quantities with well-established protocols in under laboratory conditions, and their components are less prone to cellular degradation [30]. Importantly, exosomes can affect not only neighboring cells by transporting bioactive cargo, but also cells and host tissues over a greater distance [105].

Despite the benefits of exosome use, the regulation of their content and reproducibility is important to provide successful clinical applications. The function and secretion quantity of exosomes depends on their donor. It has been shown that ASCs from obese donors and those from omental depots secrete more exosomes than lean donors and subcutaneous depots, respectively, which is reflected in the disease state [114]. However, exosome secretion can be enhanced with in vitro molecule modulators. Wang et al. showed that MSCs treated with N-methyldopamine and norepinephrine for 24 h produce three times more exosomes without altering their features [115]. The exosomal content is not random and is controlled by posttranslational modifications and specific endogenous target sequences at the stage of exosome formation, most importantly, miRNAs are the most preferred exosome loaded RNAs [116]. Nevertheless, due to the improvement in the exosome efficiency in therapy, their function can also be boosted by the engineering content modifications through genetic and chemical methods and may be used as an ideal drug delivery agent [109,117]. 

## 4. Therapeutic Potential of Adipose-Derived Stem Cells Secretome

**Figure 3:**
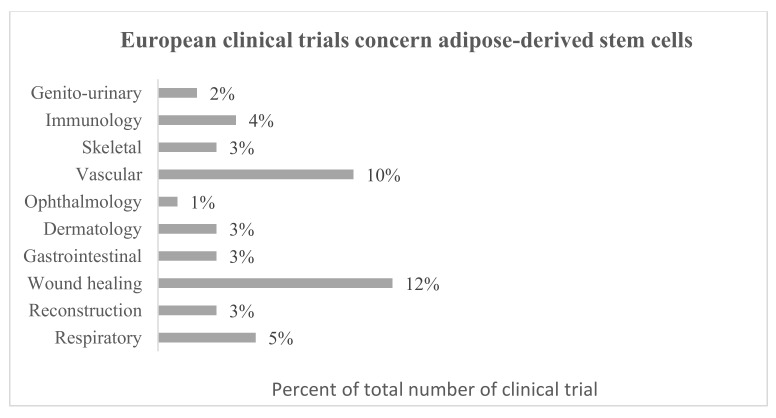
Summary of European clinical trials concern adipose-derived stem cells based on all clinical trials available in the database (years 2007–2021) [119].

**Figure 4:**
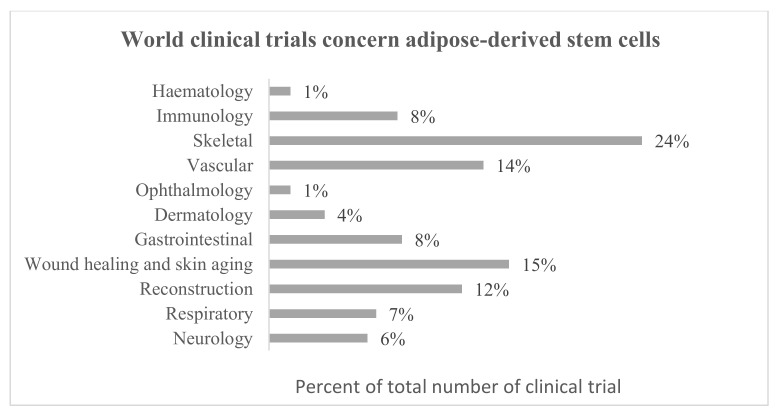
Summary of World clinical trials concern adipose-derived stem cells based on all clinical trials available in the database (years 2007–2021) [118].

Adipose-derived stem cells are getting more and more interest due to their use in regenerative medicine. Currently, 1259 MSC-containing investigational medicinal products, including 406 ASCs are registered in the Clinical Trials database [118] and also 138 and 46 in the European Clinical Trials database [119], respectively. Regarding European clinical trials database, the greatest number of ASC studies concern wound healing (fistulae, skin burns, and ulcers) and vascular applications (myocardial infarction, ischemia, and heart failure). ASCs are also studied for respiratory (respiratory distress, bacterial and COVID-19 pneumonia), immunomodulatory (HIV infection, amyotrophic lateral, and systemic sclerosis), skeletal (spinal cord injury, osteoarthritis, atrophic pseudarthrosis), dermatology (scars, cutis laxa, lichens, epidermal necrolysis), reconstructive (breasts reconstructions), gastrointestinal (hyposalivation and xerostomia), genito-urinary (urinary incontinence, erectile dysfunction), and ophthalmology (dry eye disease) applications (Figure 3). On the other hand, regarding the world Clinical Trial database, most research is focused on skeletal applications but also wound healing with the extension of research on skin aging. In this database are also registered clinical trials concerning haematological (e.g., anemia) and neurological (e.g., Parkinson’s disease) applications (Figure 4). Many clinical and laboratory research show the enormous potential of using ASCs in cell therapy (Table 1 and Table 2).

Two main perspectives are crucial in stem-cell-based therapy. First, the ability of cells, which are the progeny of implanted cells, to rebuild tissues by cell differentiation and proliferation, and second immune cells, due to their trophic activity and stimulation of endogenous regenerative factors [120]. The ASCs secretome can be obtained during culturing in vitro from the culture medium, and then conditioned medium after removal of cell debris can be used directly or fractioned as concentrated formula [121]. Below, we summarize the current stage of knowledge of ASCs and their use in neurodegenerative, inflammatory, cardiovascular, respiratory, and metabolic diseases as well as skin aging and wound healing with particular emphasis on cell-free-based therapy.

### 4.1. Neurodegenerative Diseases

Neurodegenerative diseases such as Alzheimer’s (AD), Huntington’s (HD), Parkinson’s (PD), Machado–Joseph (Spinocerebellar ataxia type 3, SCA3) diseases, and multiple sclerosis (MS) are characterized by progressive deterioration and/or death of nerve cells that disable brain function [122]. Adipose-derived stem cells secrete angiogenic and antiapoptotic factors such as VEGF, IGF [123,124], TGF-β [125], and HGF involved in neurogenic responses and tissue regeneration [33], as well as neurotrophic factors such as brain-derived neurotrophic factor (BDNF), ciliary neurotrophic factor (CNTF), glial cell line-derived neurotrophic factor (GDNF) [126,127,128], and NGF [129] are required for neurons survival and growth. Choi et al. reported that ASCs therapy in a mouse PD model restored the activity of dopaminergic neurons and mitochondrial complex I, improving the behavioral performance of mice after three weeks [130]. Recently, Park and Chang have also shown in a mouse PD model that BDNF and GDNF expressed by ASCs have an effect on the protection of dopaminergic neurons and could be used in the treatment of PD [128]. Trophic factors from ASCs can also be used in the treatment of SCA3 due to protective function of neurons by reducing the production of reactive oxygen species [131]. Furthermore, it has been proven that miR-21 from ASCs stimulates neuronal differentiation [132] as well as miR-24 [133]. 

In addition, ASC-exos show a key role in neuroprotection and neuroregeneration and have a great potential to be used in neurodegenerative disorders. It has been shown that treatment with ASC-exos can reduce amyloid beta (Aβ) levels and the Aβ42/40 ratio causing neuronal mitochondrial dysfunction in the transgenic mice-derived Alzheimer’s disease, thereby increasing cell survival and reducing neuronal death in the hippocampus and cerebral cortex [134]. ASC-exos carry enzymatically active neprilysin (NEP) that reduces the deposition of Aβ plaques in AD [135]. In turn, Lee et al. showed in in vitro mouse-derived neural cells that ASC-exos can reduce mHtt aggregate accumulation, mitochondrial dysfunction, and cell apoptosis by activating the p-CREB-PGC1α pathway and can also be used in HD treatment [136]. It has also been shown by the same authors that ASC-exos can be used in amyotrophic lateral sclerosis (ALS). Neuronal cells from the G93A ALS mouse model after treatment with ASC-exos showed a reduced level of cytosolic superoxide dismutase 1 (SOD1), which restored the abnormal reduction of mitochondrial proteins [137]. The potential for the use of ASCs extracellular vesicles in MS has also been demonstrated by Laso-Garcia et al. They have shown in a mouse model of progressive multiple sclerosis that administration of EV diminishes brain atrophy and promotes remyelination as well as reduces Th1 and Th17 levels [138]. ASC-derived exosomes used in brain-injured rats have been shown to reduce the inflammatory index (Ly6G+/CD11b/c+), as well as expression of immune cells (CD3+/CD4+/CD3+/CD8+ cells) and apoptotic cells in the circulatory system. Thereby, ASC-exos might also have the potential to prevent brain damage and neurological complications (e.g., sepsis-associated encephalopathy) that are affected by sepsis syndrome [31]. In addition, the long noncoding RNA MALAT1 from ASC-exos modulates the immune response after traumatic brain injury in vivo [139]. One Clinical Trial is currently approved to investigate the impact of ASC-exos in Alzheimer’s disease treatment (NCT04388982). The participants will be treated with exosomes from allogenic adipose-derived stem cells to cure mild to moderate dementia caused by Alzheimer’s disease at three different doses to verify their safety and efficacy. This non-randomized trial now is currently in recruitment and it is estimated to be completed in April 2022.

### 4.2. Cardiovascular Diseases

Adipose-derived stem cells are of great interest in angiogenic therapy as they are good and abundant source of proangiogenic proteins such as FGF-2, HGF, VEGF, PDGF, Ang-1, Ang-2 [33,58]. They secrete a higher concentration of Ang, LIF, and TGF-β1 factors than BM-MSCs and equal levels of VEGF-A and HGF [81]. In 2009, Wei et al. showed that concentrated ASC conditioned media can block postischemic p38 activation with concomitant neuroprotective function of IGF-1 and BDNF, thus protecting neurons in vitro and in vivo and preventing loss of hippocampal and cortical volume [140]. Similarly, Rehman et al. showed that conditioned media after ASC culture under hypoxia, administrated to mice with hind limbs showed a fivefold increased level of VEGF, and a marked improvement in perfusion [95]. The neovascularization ability and directing ASCs to the fate of vascular cells by VEGF is well known. It has been shown that after ischemia within the myocardial infarction its level increases [141]. Recently, Zhu et al. showed that extracellular vesicles from ASCs promote VEGF secretion by vascular endothelial cells in vitro and in vivo contributing angiogenesis via the let-7/argonaute 1 (AGO1)/VEGF signaling pathway [142]. In addition, Yu et al. showed that mRNA-engineered modified ASCs encoding VEGF promotes and improves vascularization and neo-angiogenesis by improving cell proliferation and vascular maturity [143]. ASC-exos have the potential to be used after ischemic stroke. Chen et al. showed that they can enhance neural regeneration and significantly reduce the infarct after ischemic stroke in a rat model. The administrated dose of ASC-exos, after the third day of acute ischemic stroke reduced cerebral edema and contraction [144]. It has been shown that ASC-exos overexpress miR-21 thus promoting vascularization of endothelial cells [30]. Interestingly, an increased level of PDGF changed the RNA and protein content in extracellular vesicles by modifying the expression of anti-inflammatory and immunomodulatory factors. These changes enhance the production of IL-10 and TGF-β1 and stimulate the formation of T-cells in order to protect the muscle from acute ischemia in vivo [145].

### 4.3. Metabolic Diseases

Cardiovascular diseases, as well as metabolic diseases (such as diabetes mellitus), may be the effect of obesity. Adipose-derived stem cells have the potential to contribute to the pathophysiology of obesity and metabolic disorders due to their bioactive secretome. Due to their immunomodulatory properties, they can regulate the metabolic inflammation during obesity, and therefore can be used in the treatment of metabolic diseases [146,147]. Moreover, ASCs isolated from WAT are desired to be used in cell therapy for metabolism disorders. They can be easily differentiated into brown adipocytes and this conversion is dependent on PPARγ agonist and differentiated cells express a higher level of uncoupling protein 1 (UCP1) important for adaptive thermogenesis. It has been already shown that ASCs-implanted animals demonstrated less weight gain than animals in the control group [146,148,149]. Recently, Ikemoto et al. showed that ASCs isolated from diabetic and non-diabetic patients exhibit the same phenotype and can be differentiated into insulin-producing cells (IPCs) characterized by the autonomous insulin secretion. They showed in a diabetic mellitus 1 mouse model that IPCs from diabetic 1 patient ASCs can affect normoglycemia through prolonged insulin secretion. Moreover, the programmed cell death ligand-1 (PDL-1) secreted by these cells can be considered as circumvention of immunity [20]. ASCs also have the potential to be used in type 2 diabetes mellitus. It has been shown that ASCs treatment improves insulin sensitivity and results in the lower number of β-cells death [21].

A similar observation was made by Cao et al. where ASC-treated mice had better glucose tolerance, reduced triglyceride, and high-density lipoprotein level as well as reduced expression of IL-6, but also increased InsR and PPARγ expression [150]. The specific properties of ASCs are related to their secretome. Zhao et al. showed that exosomes transferred to macrophages induce the anti-inflammatory M2 macrophages phenotype by STAT3 and transactivation of arginase 1 and IL-10 increasing expression of uncoupling protein 1. These results suggest that exosomes can be used in clinical treatment due to improved obesity-related inflammation and metabolism [60]. Furthermore, it has been shown that PDGF can reverse the changes induced by diabetes, such as reduced proliferation and inhibition of migration, as well as targeting sites of inflammation and impaired function of diabetic ASCs [151].

Neuropathic pain, a prevalent compilation of diabetic mellitus and it has been shown that ASCs [34] and ASC conditioned media can be used in advanced peripheral painful neuropathy [152]. Brini et al. showed that diabetic mice after ASC conditioned media (from 2 × 10^6^ ASC culture) treatment restored the balance of pro-inflammatory cytokines and prevented the loss of skin innervation. Most importantly reversed allodynia and hyperalgesia effect lasted up to 12 weeks [151]. Interestingly, the same group of Sacerdote in 2021 published a paper in which conditioned media (from 2 × 10^6^ ASCs) after injection into the tail vein in osteoarthritic mice showed rapid and lasting pain relief but did not affect tissue regeneration [153].

Exosomes have also been shown to positively affect chronic complications of diabetes mellitus such as erectile dysfunction, cognitive decline, diabetic nephropathy, or wounds. Wang et al. used exosomes isolated from ASCs in a rat model and showed that corin (transmembrane serine proteinase, converting pro-atrial natriuretic peptide (ANP) and pro-brain natriuretic peptide (BNP) to active forms) present in these exosomes has a beneficial therapeutic influence on erectile dysfunction treatment [154]. ASCs also have neuroprotective properties regardless of cause and have been shown to be of use in the prevention of cognitive impairment in diabetic mellitus patients by peripheral reduction of advanced glycation end products [155]. In turn, Jin et al. showed that ASC-exos attenuate diabetic nephropathy and reduce amount of serum creatinine, urea nitrogen, and urine protein, as well as podocyte apoptosis in a mouse model. These effects were caused by enhanced miR-486 expression and thus inhibition of the Smad1/mTOR podocyte signaling pathway [156]. On the other hand, Duan et al. showed that miR-26a-5p from ASC-exos also protects against diabetic nephropathy by targeting toll-like receptor 4 (TLR4), silencing the NF-κB pathway, and downregulating VEGF-A secretion [157]. Furthermore, Gao et al. suggest that ASC-exos renal protective effect may be through the SIRT1 signaling pathway [158]. The use of exosomes in wound healing from diabetes mellitus and other causes is described in the chapter on wound healing and skin aging.

### 4.4. Respiratory Diseases

Acute respiratory distress syndrome is often observed in critically ill patients (~10% of all patients in intensive care units worldwide), and is most commonly associated with sepsis, pneumonia (bacterial and viral), severe trauma or aspiration of gastric, and/or oral and oesophageal contents [159]. Paracrine factors such as IFN-γ, IL-1β, IL-6, IL-10, TGF-β1, TGF-6, VEGF, HGF, PGE2, Ang-1, and IDO secreted by ASCs can attenuate lung injury [160]. Shigemura et al. showed that HGF from ASCs can improve gas exchange thus treat pulmonary emphysema [161]. In turn, Kim et al. showed that ASCs nanovesicles can also inhibit pulmonary emphysema by FGF-2 dependent pathway and can be used in cell-free therapy in a lower dose than naturally delivered ASC-exos demonstrating high treatment efficiency [162]. It has been also shown that ASCs have the potential for use in acute lung injury (ALI)—severe pulmonary inflammation—treatment by increased secretion of IL-6 in anti-inflammatory modulation [163]. Recently, ASC-exos have been shown to alleviate histone-induced ALI by activating PI3K/Akt pathway. Exosomal miR-126 has been shown to be a key factor in increasing Akt phosphorylation in the mouse ALI model [80].

The national clinical database has registered one study of ASC-exos in drug resistance gram-negative bacilli pulmonary infection (NCT04544215). This randomized study with 60 participants will use exosome aerosol inhalation in two doses (8 × 10^8^ and 16 × 10^8^ for seven days) and placebo groups. The results are not yet available, the current state of this study is recruitment, and the end of the study is estimated for July 2023.

Adipose-derived stem cells are also of great interest for treatment of COVID-19 respiratory distress. It has been shown that 13 patients with COVID-19 pneumonia treated with ASCs, exhibited clinical and biological improvement and a reduction in secreted inflammatory indicators such as C-reactive protein, IL-6, ferritin, LDH, and d-dimer [164]. ASC-exos are also being tested. A clinical trial (NCT04276987) with 24 critically ill patients is testing the use of conventional treatment and ASC-exos by inhaling an aerosol five times (2 × 10^8^ nanovesicles at day 1, 2, 3, 4, 5) but the results are not yet published.

### 4.5. Skeletal Tissue Regeneration

Skeletal tissue clinical trials are registered in the highest number of all clinical trials using ASCs (Figure 4). Most of these studies focus on osteoarthritis. The ASCs secretome also has the potential to regenerate skeletal tissue. Mitchel et al. showed that ASCs secretome increases tissue regeneration and homeostasis of the mouse myoblast cell line by promoting skeletal muscle proliferation, differentiation, and migration. This may be due to the presence of soluble proteins and exosomal immunomodulatory miR-21 [165]. On the other hand, ASC-exos has been shown to induce osteogenic differentiation [166] and also suppress inflammation by upregulating miR-145 and miR-221, and therefore can be helpful in the treatment of osteoarthritis [167]. It has already been shown in the case of chondrocytes isolated from osteoarthritic patients that treatment with ASCs extracellular vesicles reduces MMP-1, MMP-3, MMP-13, and ADAMTS-5 and increases collagen II expression [168,169]. ASC-exos also have the potential to regenerate muscles. Wang et al. showed in a rat model that exosomes can prevent muscle degradation in torn rotator cuffs by atrophy, fat infiltration, inflammation, and prevention of vascularization. However, they pointed out that they had used a population of young, although mature rats, while torn rotator cuffs are common in the elderly population and that additional studies with older animals are required [170].

### 4.6. Wound Healing and Skin Aging

The skin as the first physical barrier against physical and biological damage and its self-repairing ability, protect against dehydration and thermal, chemical, and physical stress. Repairing physical damage is a dynamic process involving overlapping stages such as homeostasis, inflammation, proliferation, re-epithelization, and fibrosis [171]. ASCs secrete four major growth factors that promote re-epithelization: EGF, FGF-2, IGF-1, and TGF-β. These factors induce the necessary biological effects for tissue repair including cell migration, proliferation, and differentiation but also promotion of angiogenesis and extracellular production and inflammation resolution [101]. It has already been shown that co-treatment with administration of ASCs and conditioned ASCs media increases the proliferation of dermal keratinocytes and fibroblast in the skin [172] but also the maturation of fibroblasts associated with miR-24 upregulation [173]. Moreover, Pu et al. showed that ASCs conditioned media prevent flap necrosis after skin flap transplantation by increasing proliferation and secretion of IL-6 [174]. Similarly, Bai et al. showed that ASC-exos enhances skin flap recovery by reducing inflammation and apoptosis [175]. ASC-exos prolong the survival of vascularized composite allografts after transplantation by downregulating CD4+ T and Th1 cells and upregulating Tr1 and Treg cells [32]. It shows that the use of ASCs conditioned media as well as exosomes may be a promising approach in reconstructive and plastic surgery.

Tissue repairing requires the mitigation of inflammatory insults. Besides secreted immunomodulatory factors by ASCs, it is possible to govern inflammatory pathways by extracellular vesicles assisted by transfer at the site of injury with RNA derived from adipose-derived stem cells [105]. It has been shown that ASC-exos express miR-21, which increases migration and proliferation of HaCaT cells by enhancing the matrix metalloproteinase 9 (MMP-9) expression via the PI3K/AKT pathway, thereby increasing wound healing [176]. Furthermore, miR-19b from ASC-exos promotes wound healing by regulating the TGF-β pathway through targeting chemokine C-C motif ligand 1 (CCL1) by modulating the CCL1/TGF-β signaling axis [75]. Interestingly, TGF-β secreted by ASCs has been shown to cross react with growth differentiation factor 11 (GDF11) to reverse keratinocytes aging and trigger skin rejuvenation [177]. Improved re-epithelialization, collagen remodeling, angiogenesis, and vessel maturation and thus wound healing were also demonstrated in diabetic mice treated with engineered ASC-exos containing miR-21-5p. In Lv et al.’s study, keratinocytes proliferation and migration and accelerated wound healing were performed via the Wnt/β-catenin signaling pathway in vitro [178], confirming the results obtained by Ma et al. a year earlier, where ASC-exos was shown to improve wound healing also via the Wnt/β-catenin pathway [78]. ASC-exos can also be used as a potential factor in alleviating atopic dermatitis. It has been found that an in vivo model of atopic dermatitis after ASC-exos injection exhibited reduced clinical score, decreased level of serum IgE and blood eosinophil counts, as well as CD86+ and CD206+ cells in skin lesions, as well as diminished the infiltration of mast cells. Moreover, it has been shown that levels of inflammatory cytokines such as IL-4, IL-23, and IL-31 were reduced [179]. Recently, Shin et al. showed that not only the levels of these cytokines are reduced after ASC-exos treatment in atopic dermatitis, but also IL-5, IL-13, TNF-α, IFN-γ, IL-17, and TSLP. They also showed that after ASC-exos treatment, cells restored expression of genes responsible for lipid metabolism, the cell cycle, and the inflammatory response, as well as improving the skin barrier [180].

Furthermore, adipose-derived stem cells and their secretome can be used in skin rejuvenation and wrinkle reduction, mostly by stimulating collagen synthesis and regulating the proliferation and migration of dermal fibroblasts [181]. Guo et al. showed the protective function of ASCs conditioned media on dermal fibroblasts and keratinocytes against UVB-induced photoaging. They showed that reduced skin cellular senescence was observed in the group stimulated with conditioned media after UVB irradiation. Moreover, treatment with ASCs conditioned media improved collagen I, collagen III, elastin, and TIMP-1 expression. However, they also showed that inhibitory effect of ASCs conditioned media on human dermal fibroblasts senescence decreased with fibroblasts passages [182]. In turn, Li et al. showed that ASCs conditioned media may downregulate mitogen-activated protein kinases (MAPKs), activator protein 1 (AP-1), and nuclear factor kappa B (NF-κB) UVB-induced signaling pathways and reduce IL-6 secretion [183].

## 5. Conclusions

Adipose-derived stem cells secretome is considered as a potential agent for treatment of various diseases, e.g., multiple sclerosis, rheumatoid arthritis, osteoarthritis, fistulae, diabetes mellitus, and cardiovascular diseases, but also in skin aging and wound healing. ASCs properties are effected by the specific content of its secretome: cytokines, proteins, growth factors, and exosomes with several types of RNAs, characterized by extensive bioactivity including immunomodulatory, antiapoptotic, angiogenic, vasculogenic, neurogenic, and epithelial activity. The application of bioactive factors without administering whole cells is a safer alternative treatment and may be more effective. Nowadays, many in vitro and in vivo studies from the last four years confirm effectiveness of ASCs secretome therapy, mostly with ASCs derived exosomes. Several clinical trials are currently being evaluated for safety and effectiveness of ASC-derived exosomes therapy, but the results are not yet available. Nevertheless, further research in vivo and in vitro are needed to understand the specific role of ASCs secretome in repairing of damaged and diseased tissues.

## Figures and Tables

**Table 1:** Clinical trials of adipose-derived stem cells exosomes for cell-free based therapies. N/A—Not available [111].

NCT no.	Title	Status	Condition/ Disease	Administration	Intervention/ Treatment	Results
NCT04313647	A tolerance clinical study on aerosol inhalation of mesenchymal stem cells exosomes in healthy volunteers	Recruiting	Healthy volunteers	Aerosol inhalation	2 × 10^8^, 4 × 10^8^, 8 × 10^8^, 16 × 10^8^, 20 × 10^8^ nano vesicles/3 mL to be administrated at once to different participants sets	N/A
NCT04388982	Open-label, single-center, phase I/Ⅱ clinical trial to evaluate the safety and the efficacy of exosomes derived from allogenic adipose mesenchymal stem cells in patients with mild to moderate dementia due to Alzheimer’s disease	Recruiting	Dementia due to Alzheimer’s disease	Nasal drip	5 µg, 10 µg, 20 µg exosomes/1 mL exosomes, twice a week for 12 weeks	N/A
NCT04544215	A clinical study of allogeneic human adipose-derived mesenchymal progenitor cell exosomes (haMPC-Exos) nebulizer for the treatment of carbapenem-resistant gram-negative bacilli-induced pulmonary infection	Recruiting	Drug resistant pulmonary infection	Aerosol inhalation	8 × 10^8^, 16 × 10^8^ nano vesicles/3 mL per day for 7 days	N/A
NCT04276987	A pilot clinical study on aerosol inhalation of the exosomes derived from allogenic adipose mesenchymal stem cells in the treatment of severe patients with novel coronavirus pneumonia	Completed	Coronavirus pneumonia	Aerosol inhalation	2 × 10^8^ nano vesicles/3 mL per day for 5 days	N/A

**Table 2:** Adipose-derived stem cells’ secretome investigations for cell-free therapy.

Paracrine Factors	Model	Therapeutic Effect	References
Neurodegenerative Diseases			
BDNF, GDNF	Mouse Parkinson disease model	Protection of dopaminergic neurons.	[128]
Exosomes	Mice Alzheimer disease Model	Reduction of amyloid beta (Aβ) levels and the Aβ42/40. Increasing cell survival and decrease of neuronal death in the hippocampus and the cerebral cortex. Decrease deposition of Aβ plaques in Alzheimer Disease by exosomal neprilysin.	[134,135]
Exosomes	Mice-derived neural cells from Huntington’s disease mouse model	Reduction of accumulation of mHtt aggregates, mitochondrial dysfunction and cell apoptosis by p-CREB-PGC1α pathway activation in Huntington’s disease.	[136]
Exosomes	Mice-derived neuronal cells from Amyotrophic lateral sclerosis disease mouse model	Reduction of cytosolic superoxide dismutase 1 and restore of the abnormal reduction of mitochondrial proteins.	[137]
Exosomes	Mouse progressive Multiple Sclerosis model	Brain atrophy reduction and remyelination promotion. Reduction of Th1 and Th17 levels.	[138]
Exosomes	Brain injured male Sprague-Dawley rats	Reduction of inflammatory indicator (Ly6G+/CD11^b/c^+) and immune (CD3+/CD4+/CD3+/CD8+) cells. Reduction of early and late apoptotic cells.	[31]
Exosomes (containing or depleted of MALAT1)	Rats following a mild controlled cortical impact	Modulation of inflammation-related after traumatic brain injury by MALAT1.	[139]
Cardiovascular diseases			
Exosomes	Endothelial cells	Promotion of vascularization by overexpressing miR-21.	[30]
VEGF	Human microvascular endothelial cells and mouse Hindlimb Ischemia model	Reduction of endothelial cells apoptosis. Perfusion improvement in ischemic hindlimbs.	[95]
IGF-1, BDNF	Cerebellar granule neurons	Blocking postischemic p38 activation. Protection of neural cells.	[140]
Exosomes	Rat Acute Ischemic Stroke model	Increase of neural regeneration. Reduction of brain infarct zone, brain swelling, and shrinkage.	[144]
Exosomes released by PDGF-stimulated adipose-derived stem cells	Acute Hindlimb Ischemia mouse model	Muscle protection from Acute Ischemia.	[145]
Engineered modified exosomes encoding VEGF	Nude mouse fat transplantation model	Improvement of neo-angiogenesis and vascularization by promoting cell proliferation and vascular maturity.	[143]
Exosomes	Vascular endothelial cells and nude mice transplantation model	Promoting exogenous angiogenesis. Increase the proliferation, migration, tube formation, and VEGF secretion. Improving the survival of fat grafts.	[142]
Metabolic diseases			
PDGF	Diabetic patients adipose-derived stem cells and SCID Wound mice model	Increased proliferation, migration, and homing to sites of inflammation.	[151]
Exosomes	Diabetic rat model	Restored erectile function due to corin content in exosomes.	[154]
Exosomes	Mouse podocyte MPC5 cells and spontaneous diabetes mice	Reducing high glucose-induced increase of cell death. Reduction of urine protein, serum creatinine, blood urea nitrogen, and podocyte apoptosis.	[156]
Exosomes (miR-26a-5p)	Mouse podocyte MPC5 cells and spontaneous diabetes mice	Protection of cells from injury. Protection against diabetic nephropathy.	[157]
Exosomes	Peritoneal macrophages and obesity mouse model	Improving obesity-related inflammation and metabolism.	[60]
Exosomes	Sepsis-induced acute kidney injury mouse model	Renal Protective effect in acute kidney injury.	[158]
Respiratory diseases			
HGF	Pulmonary emphysema rat model	Increased alveolar and vascular repair.	[161]
Artifical Nanovesicles	MLE-12 epithelial cells and elastase-induced emphysema mice	Proliferation increase. Inhibition of emphysema primarily by an FGF2-dependent pathway.	[162]
Exosomes	Histone-induced acute lung injury mice	Improvement of survival. Inhibition histone-mediated lung hemorrhage edema, Reduction of vascular hyper-permeability.	[80]
Skeletal tissue regeneration			
Exosomes	Adipose-derived stem cells	Induction of osteogenic differentiation.	[166]
Exosomes	Synovial fibroblasts and periosteal cells	Chondrogenesis promotion. Increased Collagen type II and β-catenin expression. Increased proliferation (miR-145) and chondrogenic potential (miR-221).	[167]
Conditioned Media	Osteoarthritic mice model	Rapid and lasting pain relief. No effect on tissue regeneration.	[153]
Exosomes	Chondrocytes from osteoarthritic patients	Reduction of MMP-1, MMP-3, MMP-13 and ADAMTS-5. Increased collagen II expression.	[169]
Exosomes	Chondrocytes from osteoarthritic patients	Reduction of MMP-3 expression. Increased collagen II expression.	[168]
Whole secretome/isolated exosomes	Mouse myoblast cell line C2C12 and C57BL/6 mice	Increased cell proliferation, skeletal muscle differentiation and migration. Enhancing of regeneration of skeletal muscle in acute damage.	[165]
Exosomes	Massive rotator cuff tear rat model	Prevention of atrophy, fatty infiltration, inflammation, and vascularization of muscles.	[170]
Wound healing and skin aging			
Platelet-rich plasma and conditioned Media	Fibroblasts and keratinocytes isolated from skin sample	Increased cell proliferation and migration.	[172]
Conditioned Media	Human epidermal keratinocyte neonatal cells	Acceleration of keratinocyte differentiation via miR-24 upregulation.	[173]
Exosomes	Human keratinocyte cell line HaCaT	Increased migration and proliferation by exosomal miR-21. Enhancing MMP-9 expression. Promotion of wound healing.	[176]
Exosomes	Human keratinocyte cell line HaCaT	TGF-β pathway regulation by miR-19b. Promotion of wound healing.	[79]
Exosomes	Human keratinocyte cell line HaCaT	Promotion of wound healing via Wnt/β-catenin pathway. Increased proliferation and migration. Apoptosis inhibition.	[78]
Conditioned media	Human keratinocyte cell line HaCaTs and normal human dermal fibroblasts	Anti-photoaging activity. Reduced IL-6 secretion.	[183]
Engineered modified exosomes miR-21-5p	Human keratinocyte cell line HaCaT and fullthickness skin defects diabetic rat model	Promotion of wound healing via Wnt/β-catenin pathway. Improved wound healing by re-epithelialization, collagen remodeling, angiogenesis, and vessel maturation.	[178]
Conditioned Media	Ischemia/reperfusion flap mice model	Increased cell proliferation and the number of hair follicles. Prevention from flap necrosis after skin flap transplantation.	[174]
Exosomes	Human umbilical vein endothelial cell	Reduction of inflammation and apoptosis. Enhancing skin flap recovery.	[175]
Exosomes	Human embryonic kidney 293 cells	Prolonged the survival of vascularized composite allografts after transplantation. Downregulation of CD4 + T and Th1 cells. Upregulation Tr1 and Treg cell.	[32]
Conditioned media	Human dermal fibroblasts	Reduced cellular senescence of skin cells. Improved collagen I, collagen III, elastin, and TIMP-1 expression.	[182]
Exosomes	Atopic dermatitis mouse model	Decreased level of IgE and eosinophiles in blood and CD86+ and CD206+ cells in skin lesion. Reduction of (IL)-4, IL-23, IL-31.	[179]
Exosomes	Chronic allergic dermatitis mouse model	Promotion of epidermal barrier repair. Reduction of IL-5, IL-13, TNF-α, IFN-γ, IL-17, and TSLP.	[180]

## Data Availability

Not applicable.
